# On the association between chromosomal rearrangements and genic evolution in humans and chimpanzees

**DOI:** 10.1186/gb-2007-8-10-r230

**Published:** 2007-10-30

**Authors:** Tomàs Marques-Bonet, Jesús Sànchez-Ruiz, Lluís Armengol, Razi Khaja, Jaume Bertranpetit, Núria Lopez-Bigas, Mariano Rocchi, Elodie Gazave, Arcadi Navarro

**Affiliations:** 1 Unitat de Biologia Evolutiva Departament de Ciències Experimentals i de la Salut, Departament de Ciències Experimentals i de la Salut. Universitat Pompeu Fabra. Parc de Recerca Biomèdica de Barcelona. Dr. Aiguader 88. 08003 Barcelona. Catalonia, Spain; 2 Genes and Disease Program, Center for Genomic Regulation,. Parc de Recerca Biomèdica de Barcelona. Dr. Aiguader 88, 1. 08003 Barcelona. Catalonia, Spain; 3 The Center for Applied Genomics. The Hospital for Sick Children. MaRS Centre - East Tower. 101 College Street, Room 14-706. Toronto, Ontario. Canada; 4 Research Unit on Biomedical Informatics of IMIM/UPF. Parc de Recerca Biomèdica de Barcelona. Dr. Aiguader 88. 08003 Barcelona. Catalonia, Spain; 5 Dipartimento di Genetica e Microbiologia. Universita di Bari, Bari, Italy; 6 Institucio Catalana de Recerca i Estudis Avancats (ICREA) and Unitat de Biologia Evolutiva, Departament de Ciències Experimentals i de la Salut, Universitat Pompeu Fabra. Parc de Recerca Biomèdica de Barcelona. Plaça Dr. Aiguader 88. 08003 Barcelona. Catalonia, Spain; 7 CIBER Epidemiología y Salud Pública (CIBERESP), Spain; 8 Population Genomics Node (GNV8) National Institute for Bioinformatics (INB), Spain

## Abstract

**Background:**

The role that chromosomal rearrangements might have played in the speciation processes that have separated the lineages of humans and chimpanzees has recently come into the spotlight. To date, however, results are contradictory. Here we revisit this issue by making use of the available human and chimpanzee genome sequence to study the relationship between chromosomal rearrangements and rates of DNA sequence evolution.

**Results:**

Contrary to previous findings for this pair of species, we show that genes located in the rearranged chromosomes that differentiate the genomes of humans and chimpanzees, especially genes within rearrangements themselves, present lower divergence than genes elsewhere in the genome. Still, there are considerable differences between individual chromosomes. Chromosome 4, in particular, presents higher divergence in genes located within its rearrangement.

**Conclusion:**

A first conclusion of our analysis is that divergence is lower for genes located in rearranged chromosomes than for those in colinear chromosomes. We also report that non-coding regions within rearranged regions tend to have lower divergence than non-coding regions outside them.

These results suggest an association between chromosomal rearrangements and lower non-coding divergence that has not been reported before, even if some chromosomes do not follow this trend and could be potentially associated with a speciation episode. In summary, without excluding it, our results suggest that chromosomal speciation has not been common along the human and chimpanzee lineage.

## Abstract 2

Analysis of the genes located in rearranged human and chimpanzee chromosomes identified lower divergence than for those in colinear chromosomes.

## Background

Genomic DNA sequences of humans and chimpanzees differ by only 1.23% if considering only point mutations [1,2], a figure that grows up to 5% if small insertions and deletions are taken into account [3] and up to a yet unknown percentage when segmental duplications are added to the picture [2,4,5] Besides such relatively small-scale changes in their DNA sequences, the two species differ by large-scale rearrangements in their karyotypes. Human chromosome 2 results from the fusion of two acrocentric chromosomes that are independent in the great apes [6]. In addition, there are at least 7 major (larger than 10 Mb) pericentric inversions (in human chromosomes 4, 5, 9, 12, 15, 17 and 18) that range in size between 16 and 77 Mb and many smaller ones. Breakpoint regions of most of these rearrangements have been well defined both *in silico *[2,7] and experimentally [6,8-16], although the exact location of some of them is still unclear.

Over the past three years, the role that these chromosomal rearrangements might have played in the speciation processes that have separated the lineages of humans and chimpanzees has come into the spotlight. According to models of chromosomal speciation based on the recombination-reducing effects of rearrangements, genome rearrangements enhance the speciation process by limiting gene flow between the inverted chromosomes [17-20]. Under some models, such limited gene flow may preclude introgression upon secondary contact or facilitate the fixation of genes presenting geographically divergent selection [20-22]; under other models, lower gene flow may allow incompatibility genes to accumulate on different genetic backgrounds [21,23]. Under any of these models, rearranged genomic regions involved in speciation become isolated earlier compared to the rest of the genome. For pairs of species that have diverged in recent times by means of chromosomal speciation, these models predict an association between speciation-related rearrangements and higher rates of sequence divergence [20,21,23,24]. Under models based on the accumulation of incompatibilities, protein evolution rates may also be higher since amino acid changes are more likely to take part in incompatibilities and will thus present lower gene flow than synonymous changes [23]. Current evidence for or against such models is contradictory. The first studies, including our own, that made use of human and chimpanzee DNA sequence data seemed to support the existence of an association of chromosomal rearrangements with higher rates of protein and DNA sequence evolution [19,25,26]. However, these studies were seriously affected by problems such as small sample size and biases in the data that were available in the GenBank at the time [27]. More recent studies, using larger datasets, have detected opposite trends [28] or no association at all [26-28]. Also, a study based on human-chimpanzee gene expression divergence suggested that some inversions (in particular those in chromosomes 4, 5, 9, 15 and/or 16) could have been involved in the original speciation event separating the human and chimpanzee lineages [29]. Finally, an increasing amount of data coming from other species seems to fit the chromosomal speciation model. This is the case, at the moment, of studies involving such different lineages as *Drosophila*, *Anopheles*, murids, shrew or sunflowers [17,20,30-35]. So far the question thus remains unsolved: has chromosomal speciation taken place along the human and chimpanzee lineages?

This question is even more important if one considers the current uncertainty about how the split of humans and chimpanzees came about. The traditional view of allopatric speciation at the two sides of the Rift Valley has recently been challenged by several studies suggesting parapatric speciation [36] or a complex speciation process involving secondary contact [37]. Still, neither of these works has fully convinced the community [18] and it is clear that more evidence is needed. Tests of the predictions of chromosomal speciation between humans and chimpanzees may help to build the case for or against chromosomal speciation. If higher rates of sequence divergence are found in genes included in or close to rearrangements, this can be taken as indirect evidence for chromosomal speciation and trigger further research on these genomic regions. If, in contrast, these increased rates are not found, then there is no positive evidence for the hypothesis of chromosomal speciation to be sustained, even if it cannot be totally excluded.

Here we perform one such test. We revisit the issue of chromosomal speciation between humans and chimpanzees by making use of the recently available chimpanzee genome sequence [2]. Our aims are, first, to exhaustively compare rates of pairwise human-chimpanzee sequence divergence in rearranged and in colinear genomic regions and, second, to study lineage-specific divergence rates in these same regions. To do so, we made use of the sets of measures of divergence between orthologous genes in humans, chimpanzees, rats and mice (including information for coding and non-coding sequences) gathered by the Chimpanzee Genome Consortium [2].

## Results

A simple analysis of the full set of genes in autosomes showed a pattern that was exactly opposite to our expectations. Genes in rearranged chromosomes presented lower non-coding divergence (KI), synonymous substitution rates (KS) and non-synonymous divergence rates (KA) than genes in colinear chromosomes. The ratio KA/KI was also lower in genes located in rearranged chromosomes. Similarly, genes located within evolutionary inversions in rearranged chromosomes showed lower divergence, although with lower statistical support (Table 1). Multiple causes might be underlying these results, so we endeavored to control for the several factors - such as sex chromosomes or segmental duplications - that are known to affect rates of DNA sequence evolution according to their genomic location. As shown below, these factors were studied one by one and sequentially removed from further analysis.

**Table 1:** Unfiltered dataset: comparison of evolutionary rates for genes in autosomes

	Genes in rearranged versus colinear chromosomes			Genes in rearranged chromosomes: within versus outside inversions		
		
	Colinear	Rearranged	*P *value	Outside	Inside	*P *value
N	5,873	5,818		4,710	1,108	
K_I_	0.0128	0.0126	< 0.001	0.0128	0.0126	< 0.001
K_A_	0.0033	0.003	< 0.001	0.0031	0.0028	0.048
K_S_	0.0149	0.014	0.001	0.0146	0.0118	< 0.001
K_A_/K_I_	0.2535	0.2383	0.007	0.2393	0.2342	0.605

Evolutionary rates are compared for genes in colinear versus rearranged chromosomes between human and chimpanzee, and for genes in rearranged chromosomes but inside versus outside the major cytological evolutionary rearrangements between these two species. *P *values were calculated by means of permutation tests (1,000 random permutations).

### Filtering of factors affecting divergence

First, we considered sex chromosomes in detail. It has long been known that, due to the particular evolutionary dynamics of sex chromosomes [38-41], sequences linked to the X chromosome have lower divergence rates than those linked to autosomes [31,40,42]. These results are confirmed by our analysis of human-chimpanzee pairwise divergence. Genes located in the X chromosomes presented lower synonymous substitution rates (K_S_) and lower non-coding divergence (K_I_) than those in autosomes, whereas non-synonymous divergence rates (K_A_) did not differ (Table 2). Lineage-specific substitution rates (obtained from the second dataset; see Materials and methods) showed the same trends, although significance was lost is some comparisons (Table A1 in Additional data file 1). As usually done in previous studies [2,27,29,31], we removed genes linked to sex chromosomes from further analysis.

**Table 2:** Analysis of factors known to affect evolutionary rates

	HSA X versus autosomes		Segmental duplications		Telomeres versus rest of genome		Centromeres versus rest of genome		HSA19	
					
	Genes in autosomes	Genes in HSA X	Genes outside SDs	Genes within SDs	Genes outside telomeres	Genes within telomeres	Genes outside centromeres	Genes within centromeres	Genes outside HSA19	Genes within HSA19
N	11,691	434	8,431	3,260	6,627	1,804	6,165	462	5,804	361
K_I_	0.0127	0.0094	0.0127	0.0127	0.0121	0.0149	0.0121	0.0118	0.0121	0.0132
		< 0.001		0.982		< 0.001		< 0.001		< 0.001
K_A_	0.0032	0.0029	0.0031	0.0033	0.0029	0.0040	0.0029	0.0030	0.0029	0.0032
		0.129		0.048		< 0.001		0.687		0.114
K_S_	0.0145	0.0088	0.0147	0.0138	0.0129	0.0213	0.0130	0.0118	0.0127	0.0176
		< 0.001		0.002		< 0.001		0.039		< 0.001
K_A_/K_I_	0.2459	0.2987	0.2434	0.2525	0.2370	0.2669	0.2364	0.2453	0.2360	0.2422
		0.002		0.161		< 0.001		0.537		0.671

Average divergence measures are compared between genes within and outside genomic regions previously shown to be affected by processes influencing divergence rates. See text for details.

Next we dealt with segmental duplications (SDs), since they are known to be associated with higher rates of molecular evolution [31,43,44]. In the pairwise dataset, divergence rates in the non-coding regions of genes involved in SDs (either in the chimpanzee or in the human lineage) are not different from divergence rates of single-copy genes. This is also the case for K_A _and the K_A_/K_I _ratio (Table 2). Surprisingly, however, K_S _is significantly lower in genes within SDs. To explore this discrepancy with the previous literature referenced above, we split genes overlapping SDs in three main categories: those genes that overlap SDs shared by the human and the chimpanzee lineages; genes that overlap human SDs but not chimpanzee SDs; and genes that overlap chimpanzee SDs but not human SDs (Table 3). As expected, genes overlapping human SDs showed higher divergence than genes that do not overlap with SDs. On the other hand, genes overlapping chimpanzee SDs present the opposite pattern, that is, evolutionary rates are significantly lower for coding evolutionary rates. Finally, for those genes that overlap SDs and are shared by the human and chimpanzee lineages, only synonymous divergence is lower within shared SDs. This suggests that the lower rates of divergence for genes overlapping SDs that were detected in the overall analysis may be an artifact of the preliminary state of the annotation of chimpanzee SDs. At any rate, we excluded from further analysis any gene overlapping SDs.

**Table 3:** Comparison of genes overlapping segmental duplications

	Genes overlapping shared SDs			Genes overlapping human specific SDs			Genes overlapping chimp specific SDs		
			
	Genes outside SDs	Genes within SDs	*P *value	Genes outside SDs	Genes within SDs	*P *value	Genes outside SDs	Genes within SDs	*P *value
N	5,804	330		5,804	720		5,804	1,364	
K_I_	0.0121	0.0121	0.574	0.0121	0.0122	0.032	0.0121	0.0121	0.127
K_A_	0.0029	0.0030	0.502	0.0029	0.0040	< 0.001	0.0029	0.0025	0.001
K_S_	0.0127	0.0110	0.009	0.0127	0.0138	0.016	0.0127	0.0118	0.005
K_A_/K_I_	0.2360	0.2425	0.713	0.2360	0.3126	< 0.001	0.2360	0.2068	0.002

Genes in sex chromosomes, in telomeres, centromeres and chromosome 19 were removed before this analysis to avoid known confounding factors.

The chimpanzee genome project unveiled higher human-chimpanzee divergence within 10 Mb of the telomeres [2]. This effect can be detected in both the pairwise and the lineage-specific datasets (Table 2) and for both exonic and non-coding divergence. This is a particularly important factor, since nine out of the ten major rearrangements separating the two species are pericentric inversions, that is, they exclude telomeres. Thus, considering genes in telomeres might lead to under-estimation of divergence within rearrangements. To avoid such bias, genes within 10 Mb of the telomeres were removed from further analysis.

Recent evidence suggests that, just as telomeres do, centromeric and centromeric transition regions exhibit unique organizational and evolutionary characteristics [45-47]. In our pairwise dataset, genes located within 5 Mb of pericentromeric regions at each side of centromeres showed significantly lower divergence rates than genes elsewhere in the genome (Table 2). In contrast, there are no significant lineage-specific differences in substitution rates between genes located in centromeric regions and genes in other parts of the genome (Table A1 in Additional data file 1). Given these interesting but potentially confusing patterns, genes in centromeric regions were removed from our dataset.

Finally, human chromosome 19 (HSA19) has been reported to present peculiar divergence and nucleotide composition patterns [48]. Our results also pinpoint this chromosome as an outlier. All neutral divergence measures in the pairwise dataset are markedly higher in HSA19 (Table 2). Differences in lineage-specific substitution rates are not as striking. Still, significant differences for K_S _in the human and chimpanzee lineages and for K_A _in the hominid lineage can be found (Table A1in Additional data file 1). Thus, genes located in this chromosome were also removed from our dataset.

**Figure 1:**
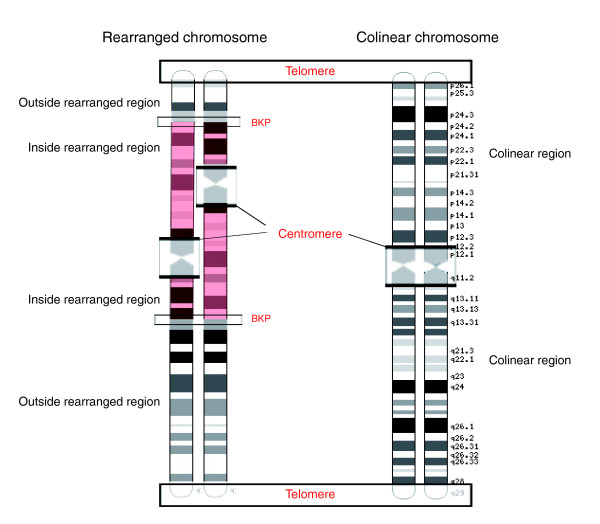
Abstract overview of the chromosomal regions that were included and excluded from our analysis. A colinear and an inverted chromosome are presented. The inversion in the rearranged chromosome is highlighted in red. For every chromosome, regions considered in this paper are labeled in black. Regions excluded from the main analysis (telomeres, centromeres and breakpoints (BKP)) are within boxes and labeled in red.

The successive removal of all the genes whose divergence values could be affected by any of the aforementioned confounding factors left 5,804 genes for pairwise analysis (dataset 1) and 2,742 in the lineage-specific analysis (dataset 2). Such filtered datasets, even if dramatically reducing our sample size, allow for a detailed testing of the hypothesis of an association between chromosomal rearrangements and genic divergence rates. A graphic overview of the regions that were included in the following analysis or excluded from it is presented in Figure 1.

### Major rearrangements

As a rough preliminary test, we repeated the comparison between rearranged and collinear chromosomes in this filtered dataset. Human-chimpanzee pairwise divergence rates are not different for synonymous sites (K_S_) or for the K_A_/K_I _ratio (Table 4). In contrast to these results and to all previous literature, average rates of non-coding, K_I_, and non-synonymous divergence, K_A_, are significantly lower in rearranged chromosomes (Table 4). That is, the original trends detected in the unfiltered dataset remain, albeit with weaker statistical support. None of the comparisons performed upon lineage-specific rates are strikingly different. Only non-synonymous divergence for humans and neutral divergence in the hominid branches present marginal differences, being lower in rearranged chromosomes.

**Table 4:** Analysis of genes according to their position in relation to rearrangements

	Genes in rearranged versus colinear chromosomes			Genes within versus outside inversions (excluding HSA2, PTR12, PTR13)			Genes outside inversions versus genes in colinear chromosomes (excluding HSA2, PTR12, PTR13)		
			
	Colinear	Rearranged	*P *value	Outside	Inside	*P *value	Colinear	Outside	*P *value
N	2,677	3,127		2,072	610		2,677	2,072	
K_I_	0.0122	0.0120	0.001	0.0120	0.0117	< 0.001	0.0122	0.0120	0.027
K_A_	0.0030	0.0028	0.036	0.0027	0.0028	0.648	0.0030	0.0027	0.014
K_S_	0.0131	0.0125	0.122	0.0127	0.0119	0.119	0.0131	0.0127	0.518
K_A_/K_I_	0.2442	0.2290	0.080	0.2255	0.2346	0.504	0.2442	0.2255	0.038

Comparison of genes in regions involved in rearrangements versus genes in colinear chromosomes or regions. Genes in breakpoints are included.

We then focused on rearranged chromosomes themselves and compared genes within inversions against genes outside them. In the pairwise dataset, non-coding sequences showed significantly lower divergence within rearrangements than outside them (0.0120 versus 0.0117, *P *value < 0.001) whereas no significant divergence differences were detected for K_A_, K_S _and the K_A_/K_I _ratio (Table 4). No general pattern was detected in the lineage-specific analysis, even if genes within rearrangements show marginally lower rates in some cases (K_A _in human branch, K_S _in the chimpanzee branch and both K_A _and K_S _in the hominid lineage; Table A2 in Additional data file 1). This suggests that the association between rearranged chromosomes and lower divergence rates reported above is mainly due to genes within the rearrangements themselves. However, when the analysis is repeated removing genes within rearrangements, divergence is still lower in genes located in rearranged chromosomes (but outside rearrangements; Table 4).

These results cannot be biased by the strict filtering applied before our main analysis. Equivalent, although stronger, trends were obtained before filtering when all genes were included in the analysis (data not shown). It is interesting, however, to consider the relative contributions of the various factors under study upon the divergence patterns between the two species. To do so, we used K_I_, since it is based on much larger amounts of data and, thus, it is less noisy than the other measures (K_I _is computed for a 250 kb window centered in each gene; see Materials and methods for details). A simple regression analysis allows us to see that, altogether, the location of genes in sex chromosomes, telomeres, centromeres, SDs, HSA19 or within rearrangements explains only about 37% of the variance in K_I _(R^2 ^= 0.372). This shows that, as expected, other smaller-scale factors, including the individual history of each gene, have a considerable influence on nucleotide divergence patterns. All the studied factors present highly significant regression coefficients (*P *values < 0.001) with the exception of centromeres, whose effect is non-significant under our linear regression model. Among the remaining factors, telomeres, HSA19 and sex chromosomes show the largest standardized regression coefficients (β = 0.488, -0.274 and 0.143, respectively; with approximately 27% of the variance explained by telomeres alone), while the fact of a gene being within rearrangements or segmental duplications has much smaller power to predict divergence values (β = -0.054 and 0.036, respectively).

### Rearrangement breakpoints

If rearrangements did affect divergence rates due to their recombination-reducing effect (including effects due to speciation-related processes), their effect should be maximum around the rearrangement breakpoints, where recombination between different chromosomal arrangements is most strongly reduced [49]. To test for this possibility, we defined windows of 2 Mb around each rearrangement breakpoint (1 Mb at each side). Then, we compared genes within these windows against all genes in rearranged chromosomes (Table 5). In the pairwise analysis, we detected lower divergence in non-coding regions surrounding the evolutionary breakpoints. Exons also show lower K_S _and K_A _values near breakpoints when compared to the rest of the chromosome, although neither of these results are statistically significant (Table 5). None of these differences can be detected in lineage-specific substitution rates (Table A3 in Additional data file 1).

**Table 5:** Comparison of genes in breakpoints versus genes in other rearranged chromosomes or regions

	Genes in breakpoints versus inverted chromosomes (excluding HSA2, PTR12, PTR13)		
	
	Rearranged	BKP	*P *value
N	2,610	72	
K_I_	0.0120	0.0113	0.001
K_A_	0.0028	0.0023	0.260
K_S_	0.0126	0.0117	0.427
K_A_/K_I_	0.2283	0.2001	0.406

BKP, breakpoint.

It would thus seem that evolutionary rates of genes close to breakpoints follow the same trend as genes within rearrangements. To check whether these two trends are independent, we removed genes surrounding breakpoints and repeated the main analysis comparing divergence within and outside rearrangements. Results did not change: in the pairwise analysis, genes within rearrangements displayed lower non-coding divergence than the rest of the rearranged chromosomes (Table 6), even if reduced sample size limits our power and some results are not significant anymore (Table A4 in Additional data file 1).

**Table 6:** Comparison of genes in regions involved in rearrangements versus genes outside inversions

	Genes within versus outside inversions (excluding breakpoints and HSA2, PTR12, PTR13)		
	
	Outside	Inside	*P *value
N	2,070	540	
K_I_	0.0120	0.0118	0.001
K_A_	0.0027	0.0029	0.301
K_S_	0.0127	0.0119	0.144
K_A_/K_I_	0.2251	0.2406	0.316

Genes in breakpoints are excluded.

Finally, the accumulation of genes with K_A_/K_S _> 1 in colinear chromosomes reported by Zhang *et al*. [28] can also be detected in our pairwise dataset, although K_A_/K_I _is used instead of the 'standard' K_A_/K_S _ratio. When focusing on rearranged chromosomes alone, no significant accumulation of genes with K_A_/K_I _> 1 was found either within or outside rearrangements (Table A9 in Additional data file 1).

### Simulated rearrangements

As explained above, genes located near the centromere had lower divergence than genes elsewhere in the genome (Table 2). This suggests that a possible explanation for our observation of lower divergence within rearrangements could be related to the fact that all the rearrangements analyzed are pericentric inversions. It is thus possible that removing genes in the centromeres and within a 5 Mb pericentromeric region on each side, as we did, is not enough to control for any potential centromere-related effects.

To test this hypothesis, we defined virtual pericentric inversions in colinear chromosomes, spanning the same average proportion of each chromosome as the real nine major inversions do in rearranged chromosomes. We compared genes within these virtual regions with genes outside them but in the same chromosomes. Table 7 shows that divergence patterns in these virtual rearrangements are similar to those in real rearranged chromosomes. In the pairwise comparison, non-coding divergence is also lower within virtual inversions (Table 7) and, again, no pattern can be detected in the lineage-specific analysis (Table A5 in Additional data file 1). This suggests that centromere-related effects extending beyond the 5 Mb windows we considered may be responsible for some, even if not all, of our observations.

**Table 7:** Comparison of genes with pericentric inversions simulated in colinear chromosomes versus genes outside them

	Genes in simulated pericentric inversions in colinear chromosomes (without HSA2 and without centromere)		
	
	Outside	Inside	*P *value
N	2,237	440	
K_I_	0.0122	0.0119	0.009
K_A_	0.0030	0.0029	0.562
K_S_	0.0129	0.0133	0.551
K_A_/K_I_	0.2448	0.2410	0.810

### Smaller rearrangements

All the above results refer to the ten major rearrangements separating humans and chimpanzees. More detailed information on the structural changes between the two species has recently become available by means of mapping chimpanzee fosmid paired-end sequences against the human genome [50]. This analysis unveiled 37 smaller rearrangements (usually < 1 Mb) which, in contrast to the major ones, do not include centromeric regions and, thus, allow the exclusion of any potential bias caused by centromeres. We compared substitution rates of genes overlapping these rearrangements with genes in colinear regions. Pairwise non-coding substitution rates were found to be marginally higher within these rearrangements (K_I _= 0.0121 versus 0.0128, *P *value = 0.020; Table 8) whereas other divergence measures do not present significant differences. This observation can not be retrieved in the lineage-specific analysis but, in any case, the sample size for this kind of approach is really small and should be treated with caution (Table A6 in Additional data file 1).

**Table 8:** Comparison of genes overlapping those inversion located *in silico *in Newman *et al*. [50]

	Genes overlapping microinversions versus genes in rest of chromosomes		
	
	Outside	Inside	*P *value
N	5,778	26	
K_I_	0.0121	0.0128	0.020
K_A_	0.0029	0.0026	0.744
K_S_	0.0127	0.0090	0.079
K_A_/K_I_	0.2362	0.2079	0.625

### Chromosome by chromosome analysis

So far, all the tests presented here were performed by pooling all rearranged chromosomes together. It is clear, however, that no chromosomal speciation model proposes that every single rearrangement ought to have played a relevant role in the speciation processes that separated humans and chimpanzees. In fact, it is reasonable to assume that most rearrangements would have appeared and become fixed along the evolutionary history of lineages (anagenesis) and not during the relatively shorter cladogenic periods [25,26]. It is thus possible that a majority of speciation-unrelated rearrangements could be masking the molecular signature of chromosomal speciation in the few rearrangements involved in such processes. Provided, of course, that there are any speciation-related rearrangements at all. In fact, a recent comparative gene-expression study hints at some chromosomes (such as HSA4, HSA5, HSA9, HSA15 and HSA16) as the most different in terms of differences in expression pattern [29].

Thus, we repeated all previous analyses on a chromosome-per-chromosome basis (Table 9; Table A7 in Additional data file 1). In most cases, the small sample size caused by our extremely conservative filtering process precludes the detection of any trend or even the performance of tests (for example, no genes from chromosomes HSA15, HSA16 or HSA 18 are included in our dataset after filtering). For the rest of the chromosomes, the trends reported after filtering were similar to those obtained with the unfiltered dataset (not shown) but, of course, lower divergence in genes within pericentric rearrangements is to be expected if, for example, the highly divergent telomeres are not filtered-out.

**Table 9:** Comparison of evolutionary rates of genes within inversions in individual chromosomes versus genes outside inversions

	Genes within versus outside inversion (no BKP 1Mb)		
	
	Outside	Inside	*P *value
**HSA1**			
N	774	6	
K_I_	0.0117	0.0111	0.207
K_A_	0.0029	0.0032	0.833
K_S_	0.0134	0.0050	0.049
K_A_/K_I_	0.2387	0.2754	0.769
			
**HSA4**			
N	183	66	
K_I_	0.0125	0.0130	0.015
K_A_	0.0030	0.0047	0.002
K_S_	0.0122	0.0120	0.896
K_A_/K_I_	0.2353	0.3468	0.017
			
**HSA5**			
N	217	105	
K_I_	0.0120	0.0120	0.950
K_A_	0.0026	0.0029	0.503
K_S_	0.0113	0.0097	0.078
K_A_/K_I_	0.2154	0.2420	0.477
			
**HSA9**			
N	197	17	
K_I_	0.0123	0.0117	0.117
K_A_	0.0027	0.0024	0.667
K_S_	0.0127	0.0135	0.750
K_A_/K_I_	0.2221	0.2016	0.777
			
**HSA12**			
N	161	170	
K_I_	0.0118	0.0115	0.013
K_A_	0.0023	0.0022	0.787
K_S_	0.0119	0.0105	0.181
K_A_/K_I_	0.1946	0.1907	0.891
			
**HSA15**			
N	195		
K_I_	0.0122		
K_A_	0.0024		
K_S_	0.0109		
K_A_/K_I_	0.1932		
			
**HSA16**			
N	219		
K_I_	0.0120		
K_A_	0.0029		
K_S_	0.0158		
K_A_/K_I_	0.2395		
			
**HSA17**			
N	40	174	
K_I_	0.0126	0.0114	< 0.001
K_A_	0.0023	0.0030	0.248
K_S_	0.0130	0.0148	0.537
K_A_/K_I_	0.1899	0.2533	0.221
			
**HSA18**			
N	72		
K_I_	0.0131		
K_A_	0.0033		
K_S_	0.0105		
K_A_/K_I_	0.2476		

Genes in breakpoints (BKP) are excluded.

HSA 4 clearly stands out in the pairwise comparison. It presents statistically higher K_A_, K_I _and K_A_/K_I _within the inversion (having removed the breakpoints). The centromeric region of HSA4 presents the usual lower divergence, thus confirming that the effect of HSA4 was not due to any special properties of its centromere extending beyond 5 Mb. In contrast to other chromosomes, genes outside the inversion in HSA4 also present higher divergence than genes in colinear chromosomes.

The other chromosome that stands out in the analysis is HSA12, which presents lower divergence, both for genes within its inversion relative to those outside it and for genes outside the inversion relative to genes in colinear chromosomes (data not shown). HSA15 presents the same trend, although with less statistical strength. Together, these two chromosomes are the major contributors to the observation of lower divergence for genes outside rearrangements than for genes in colinear chromosomes.

### Recombination rates

Recombination rates have been shown to correlate positively with divergence [51]. We first examined the relationship between recombination and the factors we have excluded from our analysis. All figures are given in cM·Mb^-1^. In our dataset, recombination rates are higher for genes located in the X chromosome than for genes elsewhere in the genome (1.43 versus 1.21, *P *value 0.027). This is also the case for genes in telomeric regions (1.09 versus 1.97, *P *value < 0.001) and in HSA19 (1.08 versus 1.57, *P *value < 0.001). All these results are congruent with previous observations [52]. Recombination rates are also lower for genes located in SDs (1.28 versus 1.04, *P *value < 0.001) and centromeric regions (1.10 versus 0.82, *P *value = 0.002).

We then focused on chromosomal rearrangements. Recombination rates for both classes of chromosomes (colinear and rearranged) are very similar (1.06 versus 1.09, *P *value not significant). Within rearranged chromosomes, recombination rates are significantly higher within inversions than in regions outside the inversion, but marginally so (1.07 versus 1.24, *P *value = 0.07). Also, regions surrounding breakpoints show higher levels of recombination than the rest of their chromosome (1.91 versus 1.08, *P *value = 0.002).

### GO categories

To see whether rearrangements were enriched in genes with functions leading to reproductive isolation, we performed an analysis of Gene Ontology (GO) [53] terms. In our dataset, several GO categories are overrepresented in rearranged regions (Table A10 in Additional data file 1). Some of the functions, such as cytokine activity, G-protein-coupled receptor binding or immune response have been previously pinpointed as enriched in genes presenting positive selection along the human lineage [2,54-56]. Interestingly, genes related to 'behavior' are also found more often within the inverted regions than expected by chance. Finally, in the specific inversion of HSA4, only the category of response to biotic stimulus is overrepresented.

## Discussion

In the present whole-genome analysis, several puzzling patterns have been detected that were not reported by previous publications. In particular, Mikkelsen *et al*. [2] performed a full-fledged descriptive analysis of the new sequence of the chimpanzee genome and, among other analyses, they tested for an increase in the rates of protein evolution of genes in rearranged chromosomes relative to genes on colinear chromosomes and of genes within the rearrangements themselves relative to genes outside them. We extended our analysis not only to the ratio of evolutionary rates, but also to individual synonymous and non-synonymous evolutionary rates. Moreover, we carefully screened rearranged and colinear regions together with their breakpoints.

A first conclusion of our analysis is that, overall, divergence is lower for genes located in rearranged chromosomes than for those in colinear chromosomes. The effect is of the same order as that of SDs. This result - consistently obtained both before and after applying any filters to our data - contradicts all previous observations. First, it contradicts the original analysis by one of us, which, based on small datasets, reported a trend for increased divergence in rearranged chromosomes [19,25,26]. And, second, it is also contrary to the results of Zhang *et al*. [28] and Vallender *et al*. [27], who found no significant association between rearrangements and average genic evolutionary rates using large datasets. Another pattern emerging from our results is that, when focusing on rearranged chromosomes, non-coding regions within rearranged regions tend to have lower divergence than non-coding regions outside them. Again, this result suggests a relationship between chromosomal rearrangements and lower non-coding divergence that has not been reported before. Moreover, this overall trend is against the general predictions of the models of suppressed-recombination chromosomal speciation and, thus, this suggests that the lineages of humans and chimpanzees have not frequently speciated by such a mechanism.

Clusters of genes under strong functional constraints located non-randomly within rearrangements might produce similar effects to those reported here. However, the finding that this association is stronger in non-coding regions than in coding regions would rule out this explanation, as coding sequences are, on average, under stronger functional constraints than non-coding regions.

But why should non-synonymous and non-coding divergence be lower in rearranged chromosomes, particularly within rearrangements? It is tempting to speculate that rearrangements tend to occur in regions with particular sequence features, such as lower recombination and, thus, lower ancestral polymorphism that would translate into lower divergence. Also, it is possible that changes in recombination rates induced by rearrangements could be affecting mutation rates. However, we lack the ancestral recombination data that would be needed to properly test these hypotheses. Extant evidence is not only scarce, but contradictory. For example, in humans there are no differences in rates of recombination between rearranged and colinear chromosomes (Table A2 in Additional data file 1), but, of course, one would not expect fixed inversions to affect current recombination rates. Evidence weakly hinting at lower ancestral polymorphism comes from current polymorphism levels in humans. Using intraspecific population data from the 256 genes in SeattleSNP [57], we found that nucleotide divergence is lower in rearranged chromosomes than in colinear chromosomes (8.13 × 10^-4 ^versus 9.34 × 10^-4^, *P *value = 0.021), but there were no differences between genes outside the rearrangements versus genes inside them (7.45 × 10^-4 ^versus 8.26 × 10^-4^, *P *value = 0.42). Still, the last analysis must be taken with care, since the number of genes within inversions was as low as 20.

Another potential explanation comes from the effect of centromeres. The major rearrangements analyzed in this paper are all pericentromeric. Even when removing genes in centromeres and within 5 Mb of pericentromeric regions, we can still see lower divergence within rearrangements. This is not the case for small inversions, which do present slightly higher non-coding divergence. Taken together, these data suggest that centromeres have a divergence-reducing effect that extends beyond 5 Mb and helps to explain our global observation. However, divergence rates are still lower for genes in rearranged chromosomes after removing genes within rearrangements, a result for which, at the moment, we lack an explanation. At any rate, these observations should be interpreted carefully, as they are based on the comparison of only two genomes. As noted by Navarro and Barton [19] and Vallender *et al*. [27], the genome-wide non-uniform distribution of genes and rates of divergence could be at the origin of our observation. Additional analyses involving more species and making use of outgroup sequences are needed to clarify this point.

As to the evolutionary rates of specific lineages, it is not surprising to find almost no significant differences. The murid lineage can not be defined as a 'close' brother lineage to the human-chimpanzee speciation, and, thus, is giving us an unbalanced tree with long inner and short terminal branches. As a consequence, we lack power in the interesting terminal branches (that is, the chimpanzee and human branches). More appropriate species for this sort of comparison will be available shortly, making it possible to increase the power of this analysis by adding density to the primate tree.

Another interesting observation is related to the relationship between recombination rates and rearrangements. We report higher recombination rates in regions surrounding evolutionary breakpoints. It is widely admitted that recombination is greatly reduced around rearrangement breakpoints of heterokaryotypic individuals [49] and this may seem to contradict our results. However, it is quite clear that measures of recombination reported here correspond to present, and not to ancestral, recombination rates. Because recombination rates change dramatically over time [58] we can not infer any relevant conclusion about this relationship. It is, however, tempting to speculate that rearrangements may tend to take place in regions of high recombination. New primate recombination data from chimpanzees and other primate species (such as Bornean and Sumatran orangutans, especially since a chromosomal inversion differentiates these two subspecies [59]) will help to shed some light on this issue.

Our final observation is that certain chromosomes seem to present some strong individual trends. Blurry results are to be expected in this analysis, since our statistical power was greatly reduced by the conservative approach we choose (outright removal of certain factors) and, thus, any putative chromosome-per-chromosome patterns are likely to be overshadowed by the great variation of rates of divergence across the genome. Analysis of unfiltered data produces the same patterns, of course, but most of the effect is due to telomeres. Still, in a general context of lower divergence within rearrangements, chromosome 4 presents significantly higher divergence rates for genes inside its inversion. This result is consistent with previous analysis of gene expression and sequence data [2,29].

An important issue is the relevance of our observations to the problem of the mode of speciation between humans and chimpanzees and along their respective lineages. Our results show that there is very little positive evidence for recurrent chromosomal speciation along the human or chimpanzee lineages. The prediction of higher DNA sequence divergence that suppressed-recombination models of chromosomal speciation make is not fulfilled by most rearrangements. However, chromosomal speciation can not be fully ruled out for several reasons. First, a chromosomal speciation episode involving HSA4 is possible, since this rearrangement harbors highly divergent genes with interesting GO functions, such as response to stimulus produced by other living organisms (biotic stimulus), which could well be related to adaptation. Second, chromosomal speciation might have taken place, but it might have been too quick or too ancient to be detected with extant sequence data. And third, speciation might have involved other functional elements besides the single-copy protein-coding genes that have been the object of all analyses published so far. These elements could be genes that do not code for proteins (microRNAs, for example); other regulatory elements (such as transcription factor binding sites) or even protein-coding genes included in SDs, which we and other authors have always filtered-out.

In the near future, it will become possible to perform detailed tests upon individual chromosomes, or rearrangements, by means of a proper set of outgroups. Also, the increasing amount of genomic information will allow us to include other functional elements in the tests. In the meantime, however, the issue of the mode of speciation between humans and chimpanzees will remain just as elusive as revealed by the recent works trying to look for signals of parapatric or allopatric speciation between the two species [18,36,37,60]. More experimental and theoretical knowledge needs to be gathered before the debate can be satisfactorily settled.

## Conclusion

Based on the observations we report here, chromosomal speciation does not appear to have been common along the human and chimpanzee lineages, although chromosome 4 clearly stands out as the best candidate to have played a role in some particular speciation process. In the future, the detailed study of the interaction of chromosomal rearrangements with some of the factors we removed in the present study, particularly with SDs, will certainly shed light on the issue of the genomic distribution of rates of genic evolution.

## Materials and methods

### Sequence gathering and evolutionary rates

All data analyzed were retrieved from the initial chimpanzee genome sequence [2] and the methods therein should be consulted. In summary, two databases were used. First, a set of more than 13,000 unambiguous human-chimpanzee orthologous genes filtered to avoid overrepresentation of gene families. From that initial dataset, only those genes with unequivocal coordinates in both species were kept. The chromosomal position of the sequences is a key parameter of our analysis, and, thus, genes in random chromosomes were also removed from our analysis, leaving a total of 12,135 genes.

For every coding sequence, several conventional indexes of molecular evolution, such as the number of non-synonymous substitutions per non-synonymous site (K_A_), the number synonymous substitutions per silent site (K_S_), and their ratio (K_A_/K_S_) were estimated using the maximum likelihood method implemented in the package PAML [61]. Substitution rates for non-coding sequence were calculated as K_I_, the number of substitutions per non-coding nucleotide. A K_I _value was obtained for a window of 250 kb, centered on each gene. We used K_A_/K_I _instead of K_A_/K_S _as the measure of rates of protein evolution, because of the close proximity between human and chimpanzees, which results quite often in a K_S _equal to 0. The averages for K_A_, K_S_, K_I_, and the ratio K_A_/K_I _are 0.00317, 0.0142, 0.0126 and 0.2483, respectively. Because of the strict criteria defined to retrieve the set of orthologous genes, the maximum values of each index are not high enough to be suspicious of false orthology or misalignment (K_S _< 0.32, K_A _< 0.055 and K_I _< 0.0259)

A second dataset was used to calculate lineage specific evolutionary rates. More than 7,000 unambiguously orthologous genes were recovered for 4 species (human, chimpanzee, rat and mouse). We applied the same filtering criteria as in the previous dataset and were left with a set of 4,905 orthologous genes with coordinates in both species and evolutionary rates for every branch in the non-rooted tree. Finally, the lineage specific evolutionary rates were estimated using a non-rooted tree in PAML.

### Polymorphism data

Polymorphism data were gathered from the SeattleSNP webpage [57]. Briefly, we downloaded nucleotide diversity measures for 256 genes. These measures have been obtained from full resequenceing of 24 African-American and 23 European (Centre d'Etude du Polymorphisme Humain (CEPH)) subjects.

### Recombination

Human recombination rates, measured in cM·Mb^-1^, were obtained from the fine-resolution recombination map in the USCS genome browser by selecting the track SNP Recombination Rates. Estimates are based on the HapMap phase I data, release 16a, and Perlegen data [62]. Fine scale recombination maps are not yet available for chimpanzees. All genes were assigned a recombination rate computed as the average of all SNPs included within them. Any genes for which recombination rates could not be determined were removed from any recombination-based analysis.

### Structural information

Coordinates of telomeres and centromeres of all chromosomes were obtained from Build 34 of the human genome [63] and NCBI Build 1 of the chimpanzee genome [63]. We considered as rearranged chromosomes all those for which major chromosomal rearrangements in either the human or the chimpanzee lineages have been indicated by recent *in silico *[2,7] or cytological data [8-13]. This comprised human chromosomes 1, 4, 5, 9,12,15, 16, 17 and 18, which differ by a pericentric inversion, and human chromosome 2, which has been generated by an ancestral telomere-telomere fusion [6]. For all chromosomes, all *in silico-*estimated coordinates were compared with newly available cytological data in order to confirm inversion coordinates. The most remarkable difference from both methodologies comes from chromosome 1, in which an inversion of about 30 Mb was detected *in silico *that has not been detected by cytological approaches (Table A9 in Additional data file 1).

#### Segmental duplications

Human and chimpanzee SD coordinates were downloaded from the Segmental Duplications Database [64,65]. As a conservative measure against false orthology, genes in our dataset overlapping the positions of SDs were removed from the analysis related to rearrangements.

### Genomic position of genes

Location information was derived from both humans and chimpanzees. When genes located in different genomic regions of interest (such as sex chromosomes, SDs or telomeres) were studied, being in one such region in either human or chimpanzee was enough to classify a gene as located in such a region. Location was established sequentially as shown in the Results section.

### Permutation tests

Genes in different categories were compared by means of pairwise permutation tests (based on 1,000 permutations). *P *values are calculated as the proportion of times that the difference of averages between two categories in a permuted dataset is equal to or larger than the observed difference.

### Go categorization and analysis

Functional annotations of genes based on GO [53] were extracted from [66] for the three ontologies Molecular function, Biological process and Cellular component. GO terms are organized into hierarchical structures such that a specialized term can be associated with several less specialized terms. We used an inclusive analysis, in which genes annotated with terms that are descendant of a term corresponding to a given level take their annotation from their parent.

To test whether there was a significant deviation from random expectation for distribution of GO annotations for genes in colinear chromosomes compared to genes in rearranged chromosomes or genes within the inverted zone compared to genes outside of the inversion, we used the Z-score transformation:

$$Z_{x}=(X-\mu_{x})/\sigma_{\bar{x}}$$

where *μ*_*x *_= mean and $\sigma_{\bar{x}}$ = standard error). $\sigma_{\bar{x}}$ was calculated as:

$$\sigma_{\bar{x}}=\sqrt{\frac{p(1-p)}{N}}$$

where *p *= proportion of genes in the category in question and *N *= number of genes in the category. If several inclusive categories were found overrepresented in the regions of study, we picked up the significant GO category with higher hierarchical level. *P *values were estimated from Z-score using the algorithm described in [67]. Only significant values after Bonferroni correction for multiple testing were considered.

## Abbreviations

GO, Gene Ontology; SD, segmental duplication.

## Authors' contributions

T. M.-B. and J. S.-R. performed the divergence analysis. L. A., and R. K. were involved in data gathering. E. G. and J. B. participated in the discussion and interpretation of results. M. R. provided cytological information of the rearrangements and dicussion of results. N. L.-B. performed the GO analysis. T. M.-B. and A N. designed the study and wrote the paper.

## Additional data files

The following additional data are available with the online version of this paper. Additional data file 1 includes analysis of lineage-specific evolutionary rates and recombination rates for factors known to affect evolutionary rates and according to their position in relation to rearrangements as well as a comparison of evolutionary breakpoints between human and chimpanzee.

## Supplementary Material

Additional data file 1 Analysis of lineage-specific evolutionary rates and recombination rates for factors known to affect evolutionary rates and according to their position in relation to rearrangements as well as a comparison of evolutionary breakpoints between human and chimpanzee. Click here for file
