# Supplementary material for: On the association between chromosomal rearrangements and genic evolution in humans and chimpanzees
Source: Genome Biol. 2007 Oct 30;8(10):R230. doi: 10.1186/gb-2007-8-10-r230 (PMC2246304; doi:10.1186/gb-2007-8-10-r230)
Supplement: Additional data file 1 — Analysis of lineage-specific evolutionary rates and recombination rates for factors known to affect evolutionary rates and according to their position in relation to rearrangements as well as a comparison of evolutionary breakpoints between human and chimpanzee. [file gb-2007-8-10-r230-S1.doc]

**Additional Table A1.**

**Analysis of factors known to affect evolutionary rates.** Divergence rates are compared between genes within or outside genomic regions previously shown to be affected by their own evolutionary dynamics. See text for details.

|  | **Centromeres *vs*. rest of genome** | | | **HSA19** | | |
| --- | --- | --- | --- | --- | --- | --- |
|  | **Genes outside Centromeres** | **Genes within Centromeres** | **P-value** | **Genes outside HSA19** | **Genes within HSA19** | **P-value** |
|  |  |  |  |  |  |  |
| N | 2742 | 213 |  | 2620 | 122 |  |
| **K_A_ (Human)** | 0.0012 | 0.0011 | 0.327 | 0.0012 | 0.0011 | 0.737 |
| **K_S_ (Human)** | 0.0065 | 0.0065 | 0.951 | 0.0064 | 0.0094 | < 0.001 |
| **K_A_ (Chimpanzee)** | 0.0012 | 0.0012 | 0.860 | 0.0012 | 0.0013 | 0.476 |
| **K_S_ (Chimpanzee)** | 0.0064 | 0.0067 | 0.645 | 0.0063 | 0.0098 | < 0.001 |
| **K_A_ (Hominid)** | 0.0128 | 0.0130 | 0.839 | 0.0125 | 0.0197 | < 0.001 |
| **K_S_ (Hominid)** | 0.0024 | 0.0023 | 0.501 | 0.0024 | 0.0025 | 0.879 |

|  | **HSA X . *vs*. Autosomes** | |  | **Segmental Duplications** | |  | **Telomeres *vs* rest of Chromosome** | | |
| --- | --- | --- | --- | --- | --- | --- | --- | --- | --- |
|  | **Genes in Autosomes** | **Genes in HSA X** | **P-value** | **Genes outside SDs** | **Genes related with SDs** | **P-value** | **Genes not in Telomeres** | **Genes in Telomeres** | **P-value** |
|  |  |  |  |  |  |  |  |  |  |
| N | 4768 | 137 |  | 3696 | 1072 |  | 2955 | 741 |  |
| **K_A_ (Human)** | 0.0012 | 0.0010 | 0.091 | 0.0013 | 0.0011 | 0.038 | 0.0012 | 0.0016 | < 0.001 |
| **K_S_ (Human)** | 0.0069 | 0.0063 | 0.388 | 0.0072 | 0.006 | < 0.001 | 0.0065 | 0.0100 | < 0.001 |
| **K_A_ (Chimpanzee)** | 0.0012 | 0.0007 | 0.009 | 0.0012 | 0.0010 | < 0.001 | 0.0012 | 0.0014 | 0.038 |
| **K_S_ (Chimpanzee)** | 0.0071 | 0.004 | 0.002 | 0.0072 | 0.0068 | 0.271 | 0.0065 | 0.0102 | < 0.001 |
| **K_A_ (Hominid)** | 0.0141 | 0.0098 | 0.001 | 0.0145 | 0.0127 | < 0.001 | 0.0128 | 0.0215 | < 0.001 |
| **K_S_ (Hominid)** | 0.0025 | 0.0017 | 0.004 | 0.0026 | 0.0021 | < 0.001 | 0.0024 | 0.0030 | < 0.001 |

**Average human recombination rates for genomic regions of interest.** Divergence rates are compared between genes within or outside genomic regions previously shown to be affected by their own evolutionary dynamics. See text for details.

|  | **HSA X**  ***vs.* Autosomes** | | **Segmental**  **Duplications** | | **Telomeres *vs*. rest of genome** | | **Centromeres *vs*. rest of genome** | | **HSA19** | |
| --- | --- | --- | --- | --- | --- | --- | --- | --- | --- | --- |
|  | **Genes in autosomes** | **Genes in HSA X .** | **Genes outside SDs** | **Genes within SDs** | **Genes outside Telomeres** | **Genes within Telomeres** | **Genes outside Centromeres** | **Genes within Centromeres** | **Genes not in HSA19** | **Genes in HSA19** |
|  |  |  |  |  |  |  |  |  |  |  |
| **N** | 11440 | 426 | 8244 | 3197 | 6474 | 1770 | 6024 | 450 | 5672 | 352 |
| **Recombination** | 1.2154 | 1.4353 | 1.28 | 1.0496 | 1.0887 | 1.9798 | 1.1081 | 0.8292 | 1.0792 | 1.5733 |
| **P-value** |  | 0.027 |  | < 0.001 |  | < 0.001 |  | 0.002 |  | < 0.001 |

**Additional Table A2.**

**Analysis of genes according to their position in relation to rearrangements.** Comparison of genes in regions involved in rearrangements *vs*. genes in colinear chromosomes or regions. Genes in breakpoints are included.

|  | **Genes in Rearranged *vs.*** | | | **Genes within *vs*. outside inversions** | | |
| --- | --- | --- | --- | --- | --- | --- |
|  | **Colinear chromosomes** | | | **(excluding HSA2, PTR12, PTR13)** | | |
|  | **Colinear** | **Rearranged** | **P-value** | **Outside** | **Inside** | **P-value** |
|  |  |  |  |  |  |  |
| **N** | 1170 | 1450 |  | 965 | 265 |  |
| **K_A_ (Human)** | 0.0013 | 0.0012 | 0.077 | 0.0012 | 0.0009 | 0.033 |
| **K_S_ (Human)** | 0.0063 | 0.0064 | 0.606 | 0.0066 | 0.0061 | 0.328 |
| **K_A_ (Chimpanzee)** | 0.0012 | 0.0011 | 0.212 | 0.0012 | 0.0009 | 0.083 |
| **K_S_ (Chimpanzee)** | 0.0064 | 0.0062 | 0.438 | 0.0064 | 0.0053 | 0.021 |
| **K_A_ (Hominid)** | 0.0127 | 0.0123 | 0.410 | 0.0126 | 0.0110 | 0.016 |
| **K_S_ (Hominid)** | 0.0026 | 0.0023 | 0.059 | 0.0024 | 0.0019 | 0.015 |

**Analysis of recombination rates of genes according to their position in relation to rearrangements.** Comparison of genes in regions involved in rearrangements *vs*. genes in colinear chromosomes or regions. Genes in breakpoints are included.

|  | **Genes in Rearranged *vs.***  **Colinear chromosomes** | | | **Genes within *vs.***  **outside inversions (excluding HSA2, PTR12, PTR13)** | | |
| --- | --- | --- | --- | --- | --- | --- |
|  | **Colinear** | **Rearranged** | **P-value** | **Outside** | **Inside** | **P-value** |
|  |  |  |  |  |  |  |
| **N** | 2610 | 3062 |  | 2022 | 601 |  |
| **Recombination** | 1.0672 | 1.0895 | 0.639 | 1.0738 | 1.2418 | 0.070 |

**Additional Table A3.**

**Comparison of genes in breakpoints *vs.* genes in other chromosomes or regions.**

|  | **Genes in breakpoints *vs.*** | | |
| --- | --- | --- | --- |
|  | **inverted chromosomes** | | |
|  | **(excluding HSA2, PTR12, PTR13)** | | |
|  | **Rearranged** | **BKP** | **P-value** |
|  |  |  |  |
| **N** | 1227 | 27 |  |
| **K_A_ (Human)** | 0.0011 | 0.001 | 0.610 |
| **K_S_ (Human)** | 0.0065 | 0.0058 | 0.644 |
| **K_A_ (Chimpanzee)** | 0.0011 | 0.0009 | 0.673 |
| **K_S_ (Chimpanzee)** | 0.0062 | 0.0069 | 0.633 |
| **K_A_ (Hominid)** | 0.0123 | 0.0127 | 0.821 |
| **K_S_ (Hominid)** | 0.0023 | 0.0020 | 0.624 |

**Comparison of recombination rates of genes in breakpoints *vs.* genes in other chromosomes or regions.**

|  | **Genes in breakpoints *vs.* inverted chromosomes**  **(excluding HSA2, PTR12, PTR13)** | | |
| --- | --- | --- | --- |
|  | **Rearranged** | **BKP** | **P-value** |
|  |  |  |  |
| **N** | 2551 | 72 |  |
| **Recombination** | 1.0896 | 1.9157 | 0.002 |

**Additional Table A4.**

**Comparison of genes in regions involved in rearrangements *vs*. genes in colinear chromosomes or regions. Genes in breakpoints are excluded.**

|  | **Genes within *vs*. outside inversions** | | |
| --- | --- | --- | --- |
|  | **(excluding breakpoints and HSA2, PTR12, PTR13)** | | |
|  | **Outside** | **Inside** | **P-value** |
|  |  |  |  |
| N | 972 | 255 |  |
| **K_A_ (Human)** | 0.0012 | 0.001 | 0.121 |
| **K_S_ (Human)** | 0.0066 | 0.0061 | 0.351 |
| **K_A_ (Chimpanzee)** | 0.0012 | 0.0009 | 0.083 |
| **K_S_ (Chimpanzee)** | 0.0064 | 0.0052 | 0.013 |
| **K_A_ (Hominid)** | 0.0126 | 0.0108 | 0.013 |
| **K_S_ (Hominid)** | 0.0024 | 0.0019 | 0.037 |

**Comparison of recombination rates in regions involved in rearrangements *vs*. genes in colinear chromosomes or regions. Genes in breakpoints are excluded.**

|  | **Genes within vs. outside inversions**  **(excluding breakpoints and HSA2, PTR12, PTR13)** | | |
| --- | --- | --- | --- |
|  | **Outside** | **Inside** | **P-value** |
|  |  |  |  |
| **N** | 2020 | 531 |  |
| **Recombination** | 1.0744 | 1.1473 | 0.424 |

**Additional Table A5. Comparison of genes within simulated pericentric inversions *vs*. genes outside them.**

|  | **Genes in simulated pericentric inversions in colinear chromosomes**  **(without HSA2 and without centromere)** | | |
| --- | --- | --- | --- |
|  | **Outside** | **Inside** | **P-value** |
|  |  |  |  |
| **N** | 989 | 181 |  |
| **K_A_ (Human)** | 0.0013 | 0.0012 | 0.731 |
| **K_S_ (Human)** | 0.0063 | 0.0059 | 0.451 |
| **K_A_ (Chimpanzee)** | 0.0013 | 0.0010 | 0.166 |
| **K_S_ (Chimpanzee)** | 0.0063 | 0.0074 | 0.149 |
| **K_A_ (Hominid)** | 0.0125 | 0.0134 | 0.367 |
| **K_S_ (Hominid)** | 0.0026 | 0.0023 | 0.285 |

|  | **Genes in simulated pericentric inversions in colinear chromosomes**  **(without HSA2 and without centromere)** | | |
| --- | --- | --- | --- |
|  | **Outside** | **Inside** | **P-value** |
| **N** | 2182 | 428 |  |
| **Recombination** | 1.0685 | 1.0607 | 0.937 |

**Additional Table A6. Comparison of genes overlapping the microinversions described by Newm*an et a*l., (2005).**

|  | **Genes overlapping microinversions *vs.* genes in rest of chromosomes** | | |
| --- | --- | --- | --- |
|  | **Outside** | **Inside** | **P-value** |
|  |  |  |  |
| **N** | 2603 | 17 |  |
| **K_A_ (Human)** | 0.0012 | 0.0012 | 0.909 |
| **K_S_ (Human)** | 0.0064 | 0.0045 | 0.301 |
| **K_A_ (Chimpanzee)** | 0.0012 | 0.0013 | 0.766 |
| **K_S_ (Chimpanzee)** | 0.0063 | 0.0040 | 0.244 |
| **K_A_ (Hominid)** | 0.0125 | 0.0078 | 0.068 |
| **K_S_ (Hominid)** | 0.0024 | 0.0026 | 0.822 |

|  | **Genes overlapping microinversions  *vs.* genes in rest of chromosomes** | | |
| --- | --- | --- | --- |
|  | **Inside** | **Outside** | **P-value** |
| **N** | 5646 | 26 |  |
| **Recombination** | 1.0795 | 1.015 | 0.875 |

**Additional Table A7.Comparison of evolutionary rates of genes within inversions *vs*. genes outside inversions in individual chromosomes. Genes in breakpoints are excluded.**

|  | **HSA1** | | | | |  | **HSA9** | | | | | |  | | **HSA16** | | | |
| --- | --- | --- | --- | --- | --- | --- | --- | --- | --- | --- | --- | --- | --- | --- | --- | --- | --- | --- |
|  | **Inside vs Outside (no BKP 1Mb)** | | | | |  | **Inside vs Outside (no BKP 1Mb)** | | | | | |  | | **Inside vs Outside (no BKP 1Mb)** | | | |
|  | **Outside** | **inside** | | **P-value** | |  | **Outside** | | **inside** | | **P-value** | |  | | **Outside** | **inside** | **P-value** | |
| **N** | 347 | 2 | |  | | **N** | 102 | | 9 | |  | | **N** | | 114 |  |  | |
| **K_A_ (Human)** | 0.0011 | 0.0000 | | 0.161 | | **K_A_ (Human)** | 0.0011 | | 0.0012 | | 0.874 | | **K_A_ (Human)** | | 0.0012 |  |  | |
| **K_S_ (Human)** | 0.0079 | 0.0153 | | 0.220 | | **K_S_ (Human)** | 0.0063 | | 0.0076 | | 0.551 | | **K_S_ (Human)** | | 0.0059 |  |  | |
| **K_A_ (Chimpanzee)** | 0.0013 | 0.0000 | | 0.289 | | **K_A_ (Chimpanzee)** | 0.0012 | | 0.0014 | | 0.849 | | **K_A_ (Chimpanzee)** | | 0.0012 |  |  | |
| **K_S_ (Chimpanzee)** | 0.0062 | 0.0000 | | 0.194 | | **K_S_ (Chimpanzee)** | 0.0072 | | 0.0076 | | 0.909 | | **K_S_ (Chimpanzee)** | | 0.0070 |  |  | |
| **K_A_ (Hominid)** | 0.0138 | 0.0111 | | 0.715 | | **K_A_ (Hominid)** | 0.0136 | | 0.0149 | | 0.748 | | **K_A_ (Hominid)** | | 0.0127 |  |  | |
| **K_S_ (Hominid)** | 0.0025 | 0.0000 | | 0.235 | | **K_S_ (Hominid)** | 0.0023 | | 0.0025 | | 0.848 | | **K_S_ (Hominid)** | | 0.0025 |  |  | |
|  | **HSA4** | | | | |  | **HSA12** | | | | | |  | | **HSA17** | | | |
|  | **Inside vs Outside (no BKP 1Mb)** | | | | |  | **Inside vs Outside (no BKP 1Mb)** | | | | | |  | | **Inside vs Outside (no BKP 1Mb)** | | | |
|  | **Outside** | **inside** | | **P-value** | |  | **Outside** | | **inside** | | **P-value** | |  | | **Outside** | **inside** | **P-value** | |
| **N** | 84 | 28 | |  | | **N** | 74 | | 71 | |  | | **N** | | 20 | 91 |  | |
| **K_A_ (Human)** | 0.001 | 0.0016 | | 0.155 | | **K_A_ (Human)** | 0.0012 | | 0.0007 | | 0.076 | | **K_A_ (Human)** | | 0.0007 | 0.0009 | 0.513 | |
| **K_S_ (Human)** | 0.0061 | 0.0048 | | 0.301 | | **K_S_ (Human)** | 0.0059 | | 0.0059 | | 0.966 | | **K_S_ (Human)** | | 0.0044 | 0.0062 | 0.366 | |
| **K_A_ (Chimpanzee)** | 0.0011 | 0.0017 | | 0.108 | | **K_A_ (Chimpanzee)** | 0.0009 | | 0.0009 | | 0.882 | | **K_A_ (Chimpanzee)** | | 0.0007 | 0.0007 | 0.947 | |
| **K_S_ (Chimpanzee)** | 0.0053 | 0.0051 | | 0.837 | | **K_S_ (Chimpanzee)** | 0.0069 | | 0.0042 | | 0.013 | | **K_S_ (Chimpanzee)** | | 0.0088 | 0.0058 | 0.105 | |
| **K_A_ (Hominid)** | 0.0107 | 0.0100 | | 0.659 | | **K_A_ (Hominid)** | 0.0121 | | 0.0093 | | 0.072 | | **K_A_ (Hominid)** | | 0.0121 | 0.0115 | 0.823 | |
| **K_S_ (Hominid)** | 0.0022 | 0.0034 | | 0.064 | | **K_S_ (Hominid)** | 0.0021 | | 0.0017 | | 0.318 | | **K_S_ (Hominid)** | | 0.0014 | 0.0017 | 0.658 | |
|  | **HSA5** | | | | |  | | **HSA15** | | | | |  | **HSA18** | | | | |
|  | **Inside vs Outside (no BKP 1Mb)** | | | | |  | | **Inside vs Outside (no BKP 1Mb)** | | | | |  | **Inside vs Outside (no BKP 1Mb)** | | | | |
|  | **Outside** | | **inside** | | **P-value** |  | | **Outside** | | **inside** | | **P-value** |  | **Outside** | | **inside** | | **P-value** |
| **N** | 109 | | 54 | |  | **N** | | 86 | |  | |  | **N** | 29 | |  | |  |
| **K_A_ (Human)** | 0.0016 | | 0.0011 | | 0.118 | **K_A_ (Human)** | | 0.0011 | |  | |  | **K_A_ (Human)** | 0.0009 | |  | |  |
| **K_S_ (Human)** | 0.0052 | | 0.0062 | | 0.374 | **K_S_ (Human)** | | 0.0066 | |  | |  | **K_S_ (Human)** | 0.0053 | |  | |  |
| **K_A_ (Chimpanzee)** | 0.0011 | | 0.0008 | | 0.262 | **K_A_ (Chimpanzee)** | | 0.0012 | |  | |  | **K_A_ (Chimpanzee)** | 0.0009 | |  | |  |
| **K_S_ (Chimpanzee)** | 0.0075 | | 0.0052 | | 0.081 | **K_S_ (Chimpanzee)** | | 0.0053 | |  | |  | **K_S_ (Chimpanzee)** | 0.0040 | |  | |  |
| **K_A_ (Hominid)** | 0.0124 | | 0.0112 | | 0.449 | **K_A_ (Hominid)** | | 0.0113 | |  | |  | **K_A_ (Hominid)** | 0.0088 | |  | |  |
| **K_S_ (Hominid)** | 0.0028 | | 0.0020 | | 0.113 | **K_S_ (Hominid)** | | 0.0024 | |  | |  | **K_S_ (Hominid)** | 0.0018 | |  | |  |

**Comparison of Recombination rates of genes within inversions vs. genes outside inversions in individual chromosomes. Genes in breakpoints are excluded.**

|  | **HSA1** | | |  |  | **HSA9** | | |  |  | **HSA16** | | |
| --- | --- | --- | --- | --- | --- | --- | --- | --- | --- | --- | --- | --- | --- |
|  | **Inside vs Outside (no BKP 1Mb)** | | |  |  | **Inside vs Outside (no BKP 1Mb)** | | |  |  | **Inside vs Outside (no BKP 1Mb)** | | |
|  | **Outside** | **inside** | **P-value** |  |  | **Outside** | **inside** | **P-value** |  |  | **Outside** | **inside** | **P-value** |
| **N** | 763 | 6 |  |  | **N** | 191 | 17 |  |  | **N** | 215 |  |  |
| **Recomb Rate** | 1.256 | 0.2597 | 0.200 |  | **Recomb Rate** | 1.268 | 1.0998 | 0.752 |  | **Recomb Rate** | 0.8088 |  |  |
|  | **HSA4** | | |  |  | **HSA12** | | |  |  | **HSA17** | | |
|  | **Inside vs Outside (no BKP 1Mb)** | | |  |  | **Inside vs Outside (no BKP 1Mb)** | | |  |  | **Inside vs Outside (no BKP 1Mb)** | | |
|  | **Outside** | **inside** | **P-value** |  |  | **Outside** | **inside** | **P-value** |  |  | **Outside** | **inside** | **P-value** |
| **N** | 182 | 66 |  |  | **N** | 159 | 168 |  |  | **N** | 39 | 169 |  |
| **Recomb Rate** | 0.9805 | 1.1491 | 0.511 |  | **Recomb Rate** | 0.8383 | 1.1926 | 0.048 |  | **Recomb Rate** | 1.1714 | 1.2167 | 0.908 |
|  | **HSA5** | | |  |  | **HSA15** | | |  |  | **HSA18** | | |
|  | **Inside vs Outside (no BKP 1Mb)** | | |  |  | **Inside vs Outside (no BKP 1Mb)** | | |  |  | **Inside vs Outside (no BKP 1Mb)** | | |
|  | **Outside** | **inside** | **P-value** |  |  | **Outside** | **inside** | **P-value** |  |  | **Outside** | **inside** | **P-value** |
| **N** | 195 | 103 |  |  | **N** | 192 |  |  |  | **N** | 72 |  |  |
| **Recomb Rate** | 0.8698 | 1.0387 | 0.369 |  | **Recomb Rate** | 0.9759 |  |  |  | **Recomb Rate** | 1.072 |  |  |

**Additional Table A8. Comparison of evolutionary breakpoints between human and chimpanzee based either in cytological or “in silico” approaches (both coordinates are based on Human assembly Build 34).**

| Cytological Approaches | |  |  | "in silico" Approach | | | |  |  |
| --- | --- | --- | --- | --- | --- | --- | --- | --- | --- |
| Hum. Chr | INV start | INV end | *Reference* | Hum. Chr | INV start | | | INV end | *Reference* |
| HSA1 |  |  |  | HSA1 | 112870424 | | | 145835091 | *10* |
| HSA2 | 114347090 | 114455823 | *1* | HSA2 |  | | |  |  |
| HSA4 | 44730692 | 86461364 | *2* | HSA4 | 44558445 | | | 86436221 | *10* |
| HSA5 | 18443766 | 96071773 | *3* | HSA5 | 18417476 | | | 95998631 | *10* |
| HSA9 | 40390489 | 84428949 | *4* | HSA9 |  | | |  |  |
| HSA12 | 20833487 | 66695639 | *5* | HSA12 | 20854309 | | | 66688318 | *10* |
| HSA15 | 17000000 | 28486050 | *6* | HSA15 | 28637194 | | |  |  |
| HSA16 | 35254239 | 46289682 | *7* | HSA16 | 35278710 | | | 46359581 | *10* |
| HSA17 | 8128215 | 48224281 | *8* | HSA17 |  | | |  |  |
| HSA18 | 5961 | 16898525 | *9* | HSA18 | 134812 | | | 16930430 | *10* |
|  |  |  |  |  |  | | |  |  |
| *1* | *Fan Y. et al, 2002. Genome Research 12:1651-1662 // Hillier, L.W., et al, 2005. Nature 434:724-731* | | | | | | | |  |
| *2* | *Kehrer-Sawatzki H. Et al, 2005. Human Mutation 25: 45-55* | | | | |  |  | |  |
| *3* | *Szamalek J.M. et al, 2005. Hum. Genet. 117: 168-176* | | | | |  |  | |  |
| *4* | *Kehrer-Sawatzki H. et al, 2005. Genomics 85: 542-550* | | | | |  |  | |  |
| *5* | *Kehrer-Sawatzki H. et al, 2005. Cytogenet. Genome Res. 108: 91-97* | | | | | |  | |  |
| *6* | *Locke D.P. et al, 2003. Genome Biol 4: R50* | | |  | |  |  | |  |
| *7* | *Goidts V. et al, 2005. Genome Res. 15: 1232-1242* | | | | |  |  | |  |
| *8* | *Kehrer-Sawatzki H. et al, 2002. Am. J. Hum. Genet. 71: 375-388* | | | | |  |  | |  |
| *9* | *Goidts V. et al, 2004. Genomics 83: 493-501* | | |  | |  |  | |  |
| *10* | *Mikkelsen, T.S et al. 2005. Nature 437: 69-87..* | | |  | |  |  | |  |

**Additional Table A9. Chi square test to detect accumulation of genes with high Ka/Ki ratios.**

| K_A_/K_I_ | Colinear Chr. | Rearrranged Chr. | Total | *Degrees of freedom: 1* |
| --- | --- | --- | --- | --- |
| < 1 | 6052 | 5666 | 11718 | *Chi-square = 11.126* |
| > 1 | 250 | 167 | 417 | *p is less than or equal to 0.001.* |
| Total | 6302 | 5833 | 12135 | *The distribution is significant.* |
|  |  |  |  |  |
|  |  |  |  |  |
|  |  |  |  |  |
| K_A_/K_I_ | Inside | Outside | Total | *Degrees of freedom: 1* |
| < 1 | 1083 | 4583 | 5666 | *Chi-square = 0.579* |
| > 1 | 28 | 139 | 167 | *For significance at the .05 level, chi-square should be greater than or equal to 3.84.* |
| Total | 1111 | 4722 | 5833 | *The distribution is not significant.* |

**Additional Table A10. Z-score to detect accumulation of genes with high Ka/Ki ratios.**

| **Enrichment of Go categories in rearranged chromosomes vs colinear chromosomes** | | | | | | | | |
| --- | --- | --- | --- | --- | --- | --- | --- | --- |
|  | | | | | | | | |
| *Gene Ontology: biological process* | | | | | | N | Z | p |
|  | | | | | |  |  |  |
| [GO:0008544 : epidermis development](http://amigo.geneontology.org/cgi-bin/amigo/go.cgi?view=details&search_constraint=terms&depth=0&query=GO:0008544&session_id=5489b1172673983&show_associations=list) | | | | | | 62 | 4.4213 | 9.81e-06 |
|  |  |  |  |  |  |  |  |  |
| *Gene Ontology: molecular function* | | | | | | | | |
|  |  |  |  |  |  |  |  |  |
| [GO:0005125 : cytokine activity](http://amigo.geneontology.org/cgi-bin/amigo/go.cgi?view=details&search_constraint=terms&depth=0&query=GO:0005125&session_id=5218b1172675513&show_associations=list) | | | | | | 109 | 5.4049 | 6.47e-08 |
| [GO:0001664 : G-protein-coupled receptor binding](http://amigo.geneontology.org/cgi-bin/amigo/go.cgi?view=details&search_constraint=terms&depth=0&query=GO:0001664&session_id=5218b1172675513&show_associations=list) | | | | | | 40 | 4.5102 | 6.47e-06 |
|  |  |  |  |  |  |  |  |  |
|  |  |  |  |  |  |  |  |  |
| **Enrichment of Go categories within INVERTED regions vs outside INVERSIONS in rearranged chromosomes** | | | | | | | | |
|  |  |  |  |  |  |  |  |  |
| *Gene Ontology: biological process* | | | | | | | | |
|  |  |  |  |  |  |  |  |  |
| [GO:0007610 : behavior](http://amigo.geneontology.org/cgi-bin/amigo/go.cgi?view=details&search_constraint=terms&depth=0&query=GO:0007610&session_id=2371b1172676237&show_associations=list) | | | | | | 82 | 4.3607 | 1.30e-05 |
| [GO:0006873 : cell ion homeostasis](http://amigo.geneontology.org/cgi-bin/amigo/go.cgi?view=details&search_constraint=terms&depth=0&query=GO:0006873&session_id=5079b1172676140&show_associations=list) | | | | | | 45 | 3.9256 | 8.60e-05 |
|  |  |  |  |  |  |  |  |  |
| *Gene Ontology: molecular function* | | | | | | | | |
|  |  |  |  |  |  |  |  |  |
| [GO:0005198 : structural molecule activity](http://amigo.geneontology.org/cgi-bin/amigo/go.cgi?view=details&search_constraint=terms&depth=0&query=GO:0005198&session_id=5486b1172676726&show_associations=list) | | | | | | 145 | 7.1604 | <1e-16 |
| [GO:0001664 : G-protein-coupled receptor binding](http://amigo.geneontology.org/cgi-bin/amigo/go.cgi?view=details&search_constraint=terms&depth=0&query=GO:0001664&session_id=1087b1172676764&show_associations=list) | | | | | | 34 | 6.75 | <1e-16 |
|  |  |  |  |  |  |  |  |  |
| *Gene Ontology: cellular component* | | | | | | | | |
|  |  |  |  |  |  |  |  |  |
| [GO:0043228 : non-membrane-bound organelle](http://amigo.geneontology.org/cgi-bin/amigo/go.cgi?view=details&search_constraint=terms&depth=0&query=GO:0043228&session_id=5878b1172677072&show_associations=list) | | | | | | 270 | 5.2615 | 1.43e07 |
|  |  |  |  |  |  |  |  |  |
| **Enrichment of Go categories within the INVERSION vs outside the INVERSION in HSA4** | | | | | | | | |
|  |  |  |  |  |  |  |  |  |
| *Gene Ontology: biological process* | | | | | | | | |
|  |  |  |  |  |  |  |  |  |
| [response to biotic stimulus ; GO:0009607](http://amigo.geneontology.org/cgi-bin/amigo/go.cgi?view=details&search_constraint=terms&depth=0&query=GO:0009607&session_id=6003b1173175204) | | | | | | 27 | 3.8341 | 1.26e-04 |
|  |  |  |  |  |  |  |  |  |
| *Gene Ontology: molecular function* | | | | | | | | |
|  |  |  |  |  |  |  |  |  |
| [receptor binding ; GO:0005102](http://amigo.geneontology.org/cgi-bin/amigo/go.cgi?view=details&search_constraint=terms&depth=0&query=GO:0005102&session_id=4979b1173175458) | | | | | | 27 | 3.8341 | 1.26e-04 |
|  |  |  |  |  |  |  |  |  |
| *Gene Ontology: cellular component* | | | | | | | | |
|  |  |  |  |  |  |  |  |  |
| [extracellular region ; GO:0005576](http://amigo.geneontology.org/cgi-bin/amigo/go.cgi?view=details&search_constraint=terms&depth=0&query=GO:0005576&session_id=4802b1173175508) | | | | | | 41 | 4.9733 | 6.58e-07 |

*N: genes with the specific Go category*

*Z: value of the transformation of the Z-score.*

*P-value: P-value of the observed Z-score compared to the random expectations.*
